# Quantifying Impact of HIV Receptor Surface Density Reveals Differences in Fusion Dynamics of HIV Strains

**DOI:** 10.3390/v17040583

**Published:** 2025-04-18

**Authors:** Anthony Gerg, Hana M. Dobrovolny

**Affiliations:** Department of Physics & Astronomy, Texas Christian University, Fort Worth, TX 76129, USA; a.d.gerg@tcu.edu

**Keywords:** HIV, syncytia formation, cytosine srabinoside, glycoproteins, surface density, density dependence

## Abstract

Human Immunodeficiency Virus (HIV) Type-1 has been studied heavily for decades, yet one area that is still poorly understood is the virus’ ability to cause cell–cell fusion. In HIV, the fusion process is mediated by viral surface glycoproteins that bind to CD4 cell receptors. This virus-mediated cell fusion creates multi-nucleated cells called syncytia that can affect infection dynamics. Syncytia formation is often studied using a cell–cell fusion assay, in which donor cells expressing the viral surface protein fuse with acceptor cells expressing the cell receptor. A mathematical model capable of reproducing the dynamics of the cell–cell fusion assay was recently developed and can be used to quantify changes in syncytia formation. In this study, we use this mathematical model to quantify the changes in syncytia formation in HIV as the surface density of the glycoproteins is varied. We find that we need to modify the model to explicitly include a density-dependent syncytia formation rate that allows us to capture the dynamics of the cell–cell fusion assay as the density of the glycoproteins changes. With this modification, we find that cell–cell fusion of the HXB2 strain, which uses the CXCR4 coreceptor, shows a threshold-like behavior, while cell–cell fusion of the Sf162 strain, which uses the CCR5 co-receptor, shows a more gradual change as surface density decreases.

## 1. Introduction

Human Immunodeficiency Virus (HIV) Type-1 is a virus that has affected millions of people around the world [1]. While HIV affects the immune system of the body by primarily infecting CD4+ T cells [2,3], it has also shown the potential to infect macrophages and dendritic cells [4,5,6,7]. HIV infection lasts a lifetime, with antiviral therapy leading to the control, but not cure, of the virus [8,9]. Due to its effects on the immune system, the body is inhibited in its response to other infections, leading to severe illness and even death [1]. For this reason, HIV has been the topic of much research in a bid to better understand the virus in its totality. Much has been discovered about HIV, including the structure of the virion [10,11], and the mechanics of fusion/entry [12], but more needs to be investigated to fully understand the infection dynamics of the virus.

**Figure 1:**
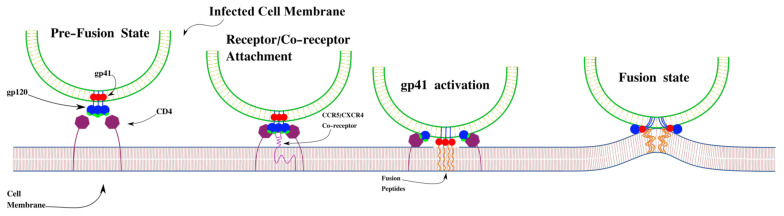
Initial fusion process of donor cells expressing the HIV Env protein and acceptor cells expressing CD4 along with either the CXCR4 or CCR5 co-receptor.

One aspect of HIV that is not well understood is virus-mediated cell fusion. The viral proteins synthesized in an infected cell play very important roles in the process of virus-mediated cell fusion [13,14,15]. These are the same proteins present on the surface of HIV virions that are responsible for fusing the viral and cell membranes. One of these proteins, gp160, must be synthesized into a glycoprotein structure consisting of gp120 and gp41 [16,17]. This glycoprotein structure, named the HIV envelope protein (Env), consists of a surface protein (gp120) and a transmembrane protein (gp41) [14], which is shown in Figure 1. The gp120 glycoprotein binds to receptor sites on healthy CD4 cells, with CCR5 and CXCR4 acting as co-receptors, starting the process of receptor-mediated fusion [18]. The co-receptor that is bound during this process—either CCR5 and CXCR4—is dependent on the strain of virus [19]. The choice of co-receptor is dependent on a component of the gp120 protein, designated as V3 (the third variable region) [20]. After binding with the CD4 receptor and co-receptor, gp120 is cleaved and then gp41 activates and inserts itself into the cell membrane, allowing for the fusion between virus and cell or between neighboring cells [13]. The resulting multi-nucleated cells, called syncytia, can continue to fuse with other uni-nuclear cells to form large structures.

HIV strains with the ability to form syncytia exhibit different infection dynamics than non-syncytia-inducing (NSI) strains [21,22,23,24,25,26,27,28]. Syncytia-inducing (SI) strains appear to lead to more rapid CD4+ T cell decline [21,25,27]. SI and NSI strains also show different preferences for the CCR5 and CXCR4 receptors [22] and differential production of certain chemokines [28]. Several studies have noted that the relative abundance of SI and NSI strains changes over the course of an HIV infection [23,24,27,29]. This can lead to the differential effectiveness of treatments on SI or NSI strains of HIV [30,31].

Yet some aspects of syncytia formation are not well understood. Much has been discovered about the biochemical reactions between the surface proteins mediating fusion, particularly the role of the density of cell surface receptors [32,33,34]. However, this quantitative understanding of the stoichiometry of viral fusion has not been extended to cell-level kinetics. Cytosine arabinoside, otherwise called AraC or cytarabine, is a transcription inhibitor that interferes with viral DNA synthesis by only allowing the early genes to be transcribed, and stopping all viral DNA synthesis after that [35]. This causes a decrease in the synthesis of the Env protein and in the overall syncytia formed. Using different doses of AraC thus allows for the regulation of the viral genetic information being transcribed, which controls the expression of viral surface proteins. Thus, the use of AraC can help lead to an understanding of how Env protein expression affects syncytia formation.

A useful tool that has been applied to virology is that of mathematical modeling. Using mathematical models can help us understand the effectiveness of the drug delivery process [36], viral load kinetics [37], and drug efficacy [38,39,40]. Mathematical modeling has also recently been used to describe syncytia dynamics and formation [41,42,43]. Two of these studies have developed mathematical models describing cell–cell fusion assays that allow us to measure syncytia formation rate and the average duration of fusion [42,43]. This methodology can be used to characterize the effectiveness of transcription inhibitors by quantifying the changes in the syncytia formation rate and the average duration of fusion as different doses of the drug are applied.

Using data from an experiment described in Lineberger et al. [32], we fit a mathematical model to cell–cell fusion assays treated with cytosine arabinoside (AraC) to determine the effect of Env protein density on cell–cell fusion. We find that we need to incorporate a density-dependent fusion rate into our model to properly explain the data.

## 2. Materials and Methods

### 2.1. Experimental Data

Lineberger et al. [32] measured the fusion of the HIV envelope protein for two different strains of HIV. The first strain (HXB2) used the gp120/gp41 virus envelope protein to bind to CD4, with the help of CXCR4 (Figure 7 in [32]). The second strain (Sf162) used the gp120/gp41 virus envelope protein to bind to CD4, with the help of CCR5 (Figure 8C in [32]). In both cases, a cell–cell fusion assay was used to examine fusion kinetics, where donor cells expressing the envelope protein were mixed with acceptor cells that express CD4 and either CXCR4 or CCR5. In this case, HeLa cells transfected using plasmids expressing the genes for creation of the gp160 envelope protein were used as donor cells. The donor cells were additionally stained with CCF2-AM, a dye that fluoresces green until it is cleaved by β-lactamase. The acceptor cells (SupT1 or SupT1/CCR5) were loaded with β-lactamase. Thus, the donor cells fluoresced green until they fused with an acceptor cell, at which point they fluoresced blue.

**Figure 2:**
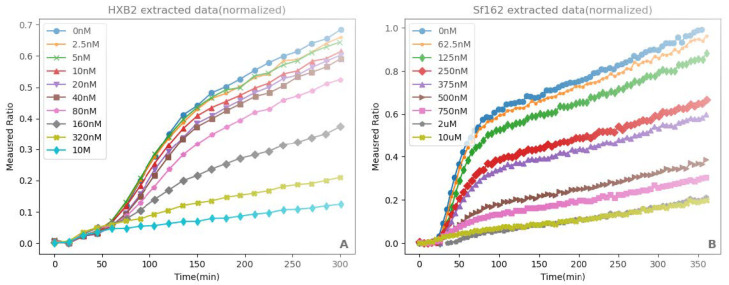
Measured blue/green ratio over time extracted for different concentrations of AraC from Lineberger et al. [32]. (**A**) HXB2 strain that uses the CXCR4 co-receptor and (**B**) Sf162 strain that uses the CCR5 co-receptor.

The cells were also treated with AraC at different doses. As discussed above, AraC is a transcription inhibitor that reduces Env protein formation. Since we used a system with AraC present in the cells, fewer Env proteins would be present on the surface, which would presumably lead to less fusion between the acceptor and donor cells. This regulation of the Env protein by AraC qualitatively changed the curves. In order to test the effect of viral envelope protein expression on fusion kinetics, the donor cells were incubated with different concentrations of cytosine arabinoside (AraC). We extracted the data regarding the blue/green ratio from Lineberger using WebPlotDigitizer (https://automeris.io/, accessed on 3 January 2024). The experimental data are shown in Figure 2.

### 2.2. Mathematical Models

We use a two-stage fusion model of the cell–cell fusion assay introduced in [42].

(1) $$\begin{array}{ccc} \frac{dD}{dt} & = & -\gamma DA \end{array}$$

(2) $$\begin{array}{ccc} \frac{dA}{dt} & = & -\gamma DA-\gamma SA \end{array}$$

(3) $$\begin{array}{ccc} \frac{dF_{1}}{dt} & = & 2\gamma DA+\gamma SA-kF_{1} \end{array}$$

(4) $$\begin{array}{ccc} \frac{dF_{2}}{dt} & = & kF_{1}-kF_{2} \end{array}$$

(5) $$\begin{array}{ccc} \frac{dS}{dt} & = & kF_{2}. \end{array}$$

**Figure 3:**
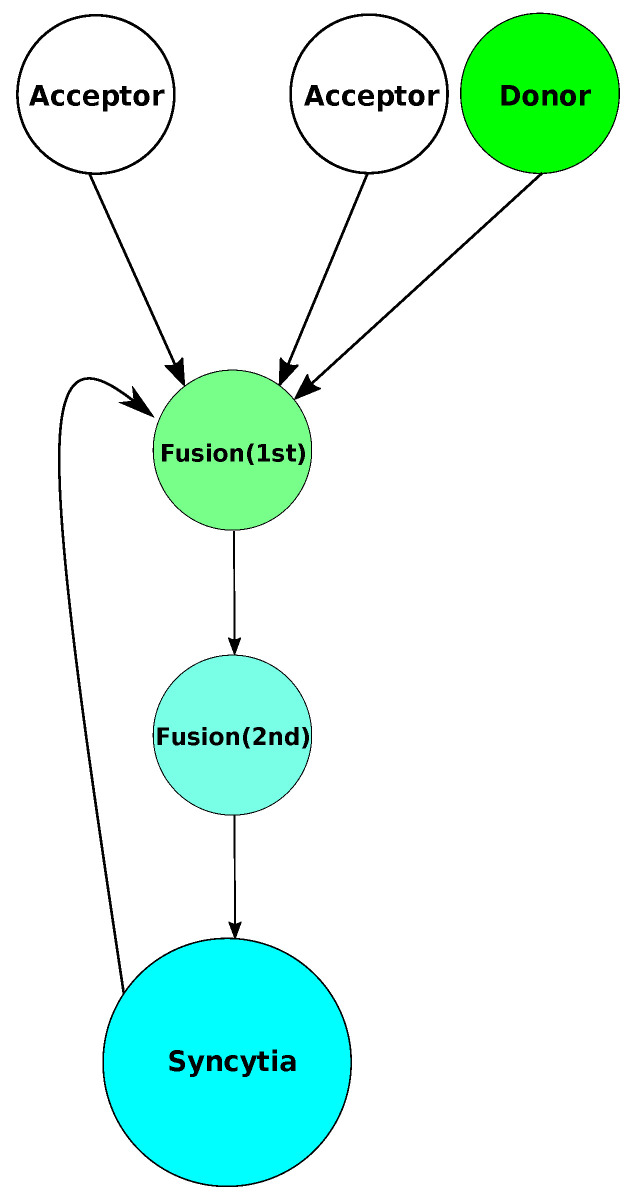
Syncytia can be formed by acceptor and donor cells starting the fusion process, entering the fusion compartments, and becoming syncytia. This is also true of previously formed syncytia and acceptor cells. Donor cells initially fluoresce in green. Once they fuse, the β-lactamase contained in acceptor cells will cause the dye to fluoresce in blue.

Equations (1)–(5) describe the changes in the types of cells within the cell–cell fusion assay. This system starts off by modeling the process of either the donor (*D*) and acceptor cells (*A*) or syncytia (*S*) and the acceptor cells (*A*) fusing together at formation rate γ. These cells then enter the fusion compartments. We use two fusion compartments, giving us an Erlang distribution for the fusion time [42]. Note that when a donor cell and an acceptor cell start to fuse, we have 2 new cells in the fusion compartment. After an average time 1/2k, they will leave the first fusion compartment ($F_{1}$) and enter the second fusion compartment ($F_{2}$). Again, after an average time 1/2k, the fusing cells leave the second fusion compartment and are finally syncytia. The entire process is shown in Figure 3.

We also investigated a model that incorporates the density-dependent syncytia formation rate. This density dependence allows for lower syncytia formation when a larger amount of syncytia are present. This is due to a smaller probability of fusion between the donor and acceptor cells as they become separated by regions of syncytia. We introduce another free parameter to represent the strength of density dependence, α. Density-dependent syncytia formation rate changes our ODEs, as follows:

(6) $$\begin{array}{ccc} \frac{dD}{dt} & = & -\frac{\gamma DA}{1+\alpha S} \end{array}$$

(7) $$\begin{array}{ccc} \frac{dA}{dt} & = & -\frac{\gamma DA}{1+\alpha S}-\frac{\gamma SA}{1+\alpha S} \end{array}$$

(8) $$\begin{array}{ccc} \frac{dF_{1}}{dt} & = & \frac{2\gamma DA}{1+\alpha S}+\frac{\gamma SA}{1+\alpha S}-kF_{1} \end{array}$$

(9) $$\begin{array}{ccc} \frac{dF_{2}}{dt} & = & kF_{1}-kF_{2} \end{array}$$

(10) $$\begin{array}{ccc} \frac{dS}{dt} & = & kF_{2}. \end{array}$$

### 2.3. Model Fitting

We characterize the system by estimating model parameter values via parameter fitting. One of the parameters is the initial (t=0) amount of donor cells present in the system, denoted by D(0). Another parameter of interest is the syncytia formation rate (γ). This parameter characterizes how often two cells will fuse once they are in contact. The final estimated parameter is that of fusion time (1/k). Fusion time governs the amount of time it takes for two cells to undergo the fusion process. We fit the models to the data by minimizing the SSR, as follows:

(11) $$SSR=\sum_{\left(i=1\right)}^{n}{\left(y_{i}-f\left(t_{i}\right)\right)}^{2},$$

where $y_{i}$ is the data measured, and $f(t_{i})$ is the model’s predicted value at $t_{i}$. In the experiment, Lineberger et al. [32] presented the measurements as ratios of blue to green fluorescence. Recall that syncytia will fluoresce in blue, and unfused donor cells fluoresce in green. We will assume that only the cells that have fully fused in our model will fluoresce in blue. Donor cells will fluoresce in green, while acceptor cells do not fluoresce. Thus, the blue to green fluorescence ratio is given by the following equation:

(12) $$f{\left(t_{k}\right)}_{i}=\frac{S(t_{k})}{D(t_{k})}.$$

Lineberger et al. stated that the blue fluorescence intensity is proportional to the area covered by syncytia. We assume that the area covered by cells is proportional to the number of cells, so fluoresence intensity is also proportional to the number of cells. We use a relative measure of the number of cells such that the total number of cells in the system is 1. This means that we assume that the initial number of acceptor cells is A(0)=1−D(0), and the initial number of cells in all other compartments is 0. Integration of the model is carried out using Python’s odeint, and minimization is performed using the Nelder–Mead algorithm in the scipy.optimize library in Python.

Akaike’s Information Criterion is used to compare model fits.

(13) $$AIC=n\log\frac{SSR}{n}+2b,$$

where *n* is the length of our dataset, SSR is our value found above, and *b* is the number of free parameters in the model. AIC becomes especially important when comparing models with different numbers of parameters. AIC penalizes for more parameters, with the model producing the lowest AIC considered the best explanation for the data.

The confidence intervals are found with bootstrapping, using 1000 surrogate datasets. Bootstrapping is performed via the following process: Take the residual values for each individual point, shuffle these residual values, and add them to the predicted best-fit values to create new datasets that have the same SSR as the original data. We use these surrogate datasets to obtain new parameters and, in doing so, we find the estimated distributions for each of the parameters. All the codes, which were created in Python 3, can be found on GitHub via the following link: https://github.com/anthony-gerg/HIV-Modeling (accessed on 15 April 2025).

## 3. Results

### 3.1. Basic Model

**Figure 4:**
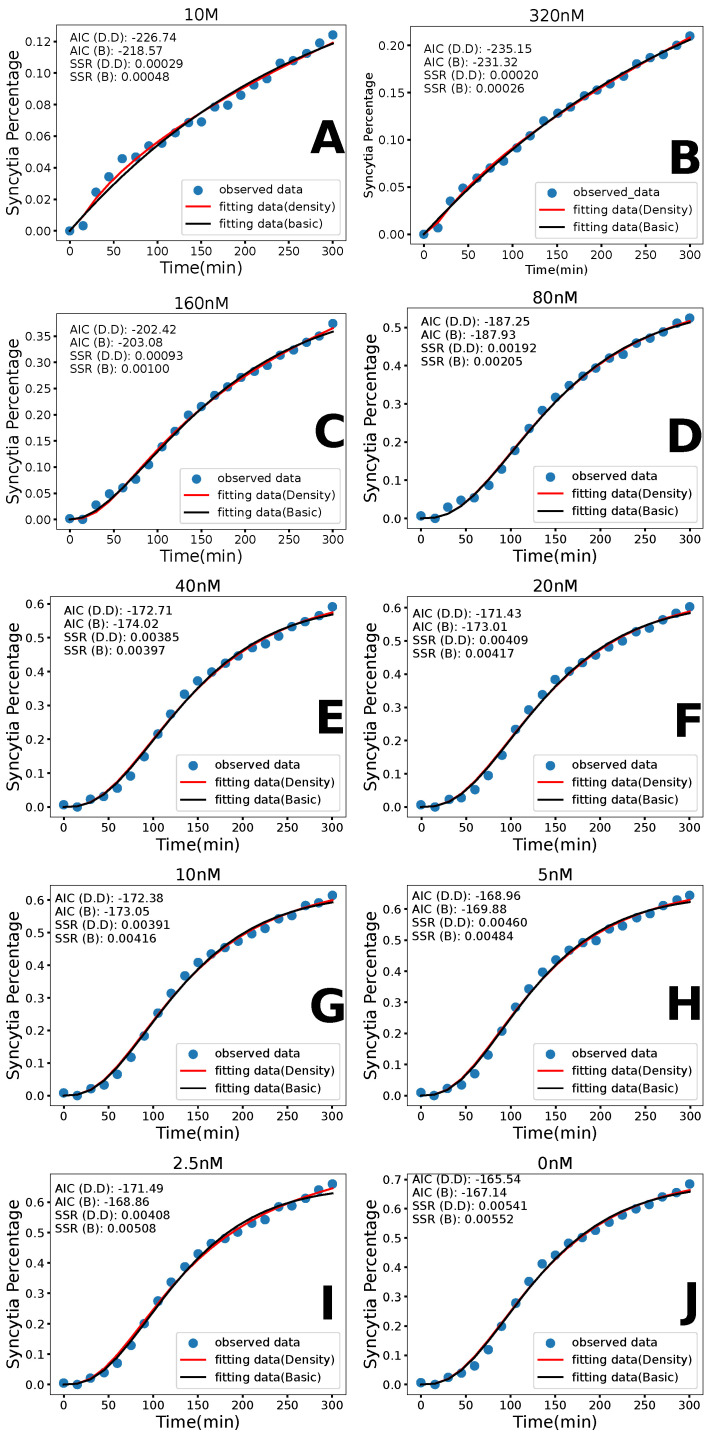
Experimental data and model fits for the HXB2 strain of HIV. Experimental data are given by the blue points. The best fit line for the basic model is in black, while the best fit line for the density dependent model is in red. Each figure (**A**–**J**) shows the results for a particular dose of AraC.

**Figure 5:**
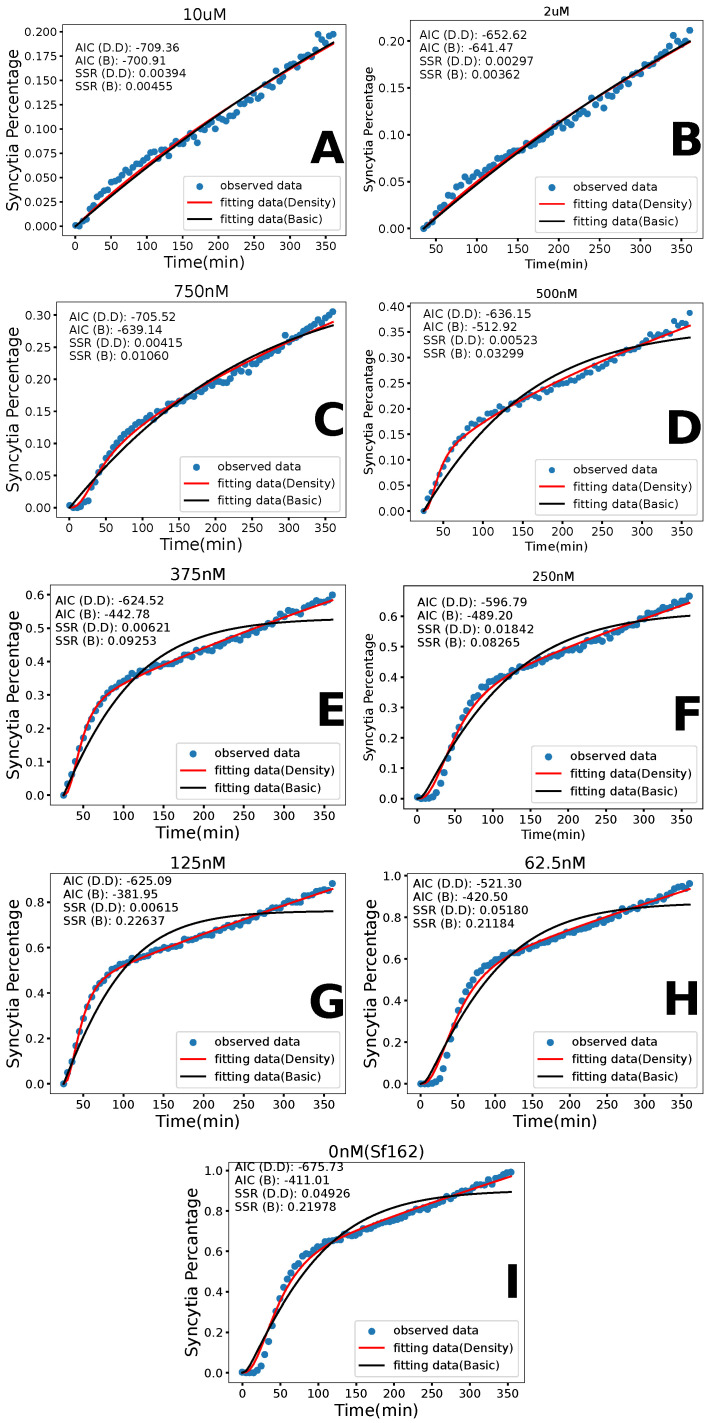
Experimental data and basic model fits for the Sf162 strain of HIV. Experimental data are given by the blue points. The best fit line for the basic model is in black, while the best fit line for the density dependent model is in red. Each figure (**A**–**I**) shows the results for a particular dose of AraC.

We first fit the data using the two-step fusion model without density-dependent syncytia formation. The experimental data along with best model fits to the data are shown in Figure 4 for the HXB2 strain and in Figure 5 for the Sf162 strain. Best-fit parameters along with 95% confidence intervals are presented in Table 1 (HXB2) and Table 2 (Sf162). Parameter correlation plots are included in the Supplementary Materials.

The basic model fits have some issues with the overall goodness of fit. The model fits the HXB2 data well (Figure 4), capturing most of the curve and resulting in a low SSR. The same cannot be said for the model fit to the Sf162 data (Figure 5). The SSRs for the Sf162 fits are much higher than the SSRs for HXB2. Qualitatively, the curvature of the fit peaks later than the experimental data points and typically misses the later time points of the data. We also note that many of the estimates for the duration of fusion are unrealistically short, which again suggests that we are not capturing the correct reason for the shape of the curve. This led us to examine the density dependence of the syncytia formation rate to possibly model the more complex curve found in the Sf162 data.

### 3.2. Density-Dependent Syncytia Formation

In the density-dependent syncytia formation rate model, we see a significant improvement in the overall fits. These fits can be seen in Figure 4 for the HXB2 strain and in Figure 5 for the Sf162 strain. The best-fit parameter values for HXB2 and Sf162 are found in Table 3 and Table 4, respectively. The parameter correlation plots are included in the Supplementary Materials.

Visually, this model fits the experimental data much better than the basic model, particularly for Sf162 strain. The SSR confirms the better fit, with the density-dependent model having a lower SSR than the basic model in all scenarios. We observe some general trends for the model parameters. For both strains of HIV, we see that the syncytia formation rate (γ) and the duration of fusion (1/k) decrease with the increasing concentration of AraC beyond some threshold concentration of AraC. Density dependence (α) increases as the concentration of AraC increases. Recall that AraC reduces the density of the Env protein on the surface of the donor cells and, by extension, also reduces the density of the Env protein on the syncytia once they are formed. This should make it harder to form syncytia, which would be consistent with a lower syncytia formation rate and increased density dependence.

### 3.3. Model Comparison

While the lower SSR suggests a better fit with the density-dependent syncytia formation rate model, this model has one more parameter than the basic model, so a direct comparison using only the SSR can be misleading. Akaike’s Information Criterion (AIC) takes into account the number of free parameters used to fit each model to the data, penalizing models with more free parameters. Table 5 shows the AIC values for both models and both strains of HIV, with the lower (better fit) AIC in bold. For the Sf162 strain, the density-dependent syncytia formation rate model has a lower AIC for all concentrations. For the HXB2 strain, the basic model essentially fit the data as well as the density dependent syncytia formation rate model (values of AIC are within 1–2 of each other) for all AraC concentrations up to 160 μM. For the two highest araC concentrations, the density-dependent syncytia formation rate model provides the better fit. At higher drug concentrations, there is a lack of Env proteins, so the density dependence is likely more apparent at these concentrations.

### 3.4. Parameter Dose Dependence

We include histograms of the parameter distributions for the density dependent model in the Supplementary Materials. For the Sf162 strain, we note that many of the parameter estimates have distinct parameter distributions that do not overlap for different drug concentrations. This suggests that the parameter value is changed by the presence of AraC. Many of the parameter distributions for the HXB2 strain overlap, with the exception of the two highest drug concentrations. This suggests that HXB2 is less affected by low doses of AraC.

**Figure 6:**
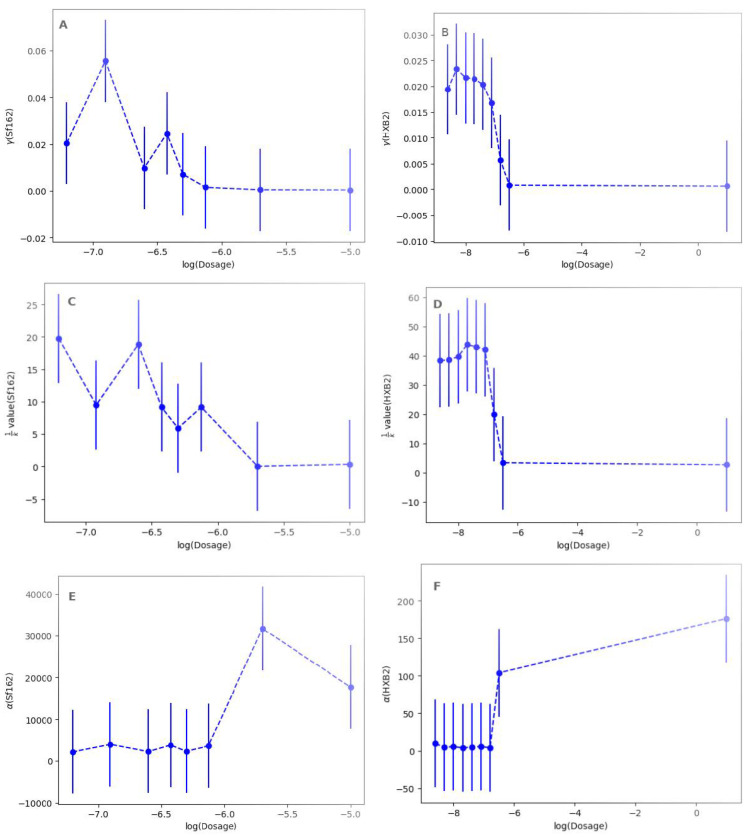
Dose–response curves for (**A**,**B**) syncytia formation rate, (**C**,**D**) fusion duration, and (**E**,**F**) density dependence for the Sf162 strain (**A**,**C**,**E**) and the HXB2 strain (**B**,**D**,**F**).

We can also consider the dose–response curves to gain further insight into how the parameters change with the AraC concentration (Figure 6). What is notable is the relative smoothness in HXB2 compared to Sf162. In Sf162, we see γ and 1/k values that follow an overall trend downwards while the density dependence increases with dosage, with a noticeable jump at dosages of 320 nM and beyond. For HXB2, the increase is much more abrupt.

## 4. Discussion

In this study, we used mathematical modeling to assess the effect of AraC on various aspects of syncytia formation in HIV. AraC is thought to reduce the density of surface proteins on the surface of the cell, making cell–cell fusion less likely. We found that we needed a model that includes a density-dependent syncytia formation rate in order to explain the data, particularly for the Sf162 strain of HIV. Specifically, we found that syncytia formation rate and fusion time decreases with increases in AraC dosage. With the density-dependent model, the density-dependent parameter α increases with increasing dosage. All of these changes are consistent with the idea that AraC decreases the density of surface proteins. Using only a visual inspection of the syncytia formation time courses, Lineberger et al. [32] concluded that while there was a decrease in the amount of fusion with an increase in AraC, there was no change in the kinetics of fusion. This is inconsistent with our results that suggest that there are, in fact, changes in the kinetics, since we observed changes in the syncytia formation rate and the fusion duration as the AraC concentration changed. Without a quantitative assessment, Lineberger et al. determined that the density of surface receptors does not change syncytia formation rate but acts as a threshold, with some minimum density required to activate fusion. Using mathematical modeling to quantify changes in the syncytia formation rate shows that there is in fact a decrease in syncytia formation rate.

As noted by Lineberger et al. [32], the kinetics of fusion appear to differ for the HXB2 and Sf162 strains. HXB2 shows a more gradual rise in the number of syncytia, while Sf162 has a rapid rise followed by slower growth in the number of syncytia. Our best-fit model parameters for the two strains have similar syncytia formation rates (γ) and similar fusion durations (1/k), but they differ in the strength of the density dependence (α), with HXB2 being more sensitive to this effect than Sf162. This is consistent with the observed behavior—HXB2’s more gradual rise is indicative of a syncytia formation rate that is slowed due to the presence of syncytia, whereas Sf162 shows a more rapid rise that is unaffected by the presence of syncytia until the number of syncytia is quite large. This could be due to the differences in interaction of CCR5 and CXCR4 with CD4 [44].

Other studies have investigated the effect of density of surface glycoproteins on syncytia formation. A similar study supports the idea that inhibitors of glycoproteins regulate syncytia formation and viral infectivity, but the overall amount of virus produced in the infection remains the same [45]. Some studies have suggested that a minimal threshold of surface proteins is required for fusion [33,34], which is more consistent with the dynamics of the HXB2 strain in our study. The HXB2 strain, which uses gp120/gp41 virus envelope protein for binding to CXCR4, shows a sharp decline in syncytia formation at an AraC dose of about 160 μM. The Sf162 strain, which uses gp120/gp41 for binding to CCR5, appears to have a more gradual decline as the dose of AraC changes. Desantis et al. were able to measure the actual density of gp120 on the surface of HIV virions [46] and found a gradual increase in infectivity as density increased, in line with our findings for the Sf126.

Another important aspect of this study is the expression and synthesis of these glycoproteins. In an infected cell, the precursor protein gp160 travels to the Golgi apparatus for folding and transcription and then heads to the granules for expression on the cell membrane [47]. Once expressed on the surface, the structure of the cell membrane allows for the movement of these Env structures into clusters. In previous research, it was found that HIV virions could not fuse as efficiently with cells if there were insufficient Env proteins in a cluster, if the cells were immature (due to random distribution of Env), or if the structure of the Env protein was altered, leading to fewer clusters of gp120/gp41 [48]. These Env clusters also enable more effective cell–cell fusion [49].

HIV is not the only virus for which fusion is dependent on the surface density of receptors. A number of studies have looked at the role of hemagglutinin surface density in the fusion of influenza [50,51,52]. Influenza also appears to have a decline in its ability to fuse as the surface density of receptors decreases [50,51] and one study noted an increase in the time to fuse [52]. Interestingly, one study using the Ebola virus suggested that very high levels of surface glycoproteins could also interfere with the fusion process [53].

While mathematical modeling is a useful tool for quantifying dynamic changes caused by drugs, every mathematical model has assumptions and limitations. One of the biggest limitations of our model is that it does not explicitly consider spatial effects. Using ordinary differential equations (ODEs) means that we assume a homogeneous mixture of our donor and acceptor cells, when in reality that is certainly not true, particularly at the later time points [54]. While the addition of density dependence is meant to at least partially account for this, it is not as accurate as explicit spatial models. Even the density-dependent model does not explicitly capture some possible spatial effects. For example, if cells happen to be on top of each other, they could potentially fuse vertically and be counted as a single cell, rather than two cells in the syncytium as assumed by the model. Agent-based models [55,56,57], which model individual cells and their interactions, can be used to account for spatial effects, although these models are computationally expensive [55]. A final limitation in our model is the lack of compensation in regards to syncytia size. As seen in the model, syncytia can fuse again after initial formation, this means syncytia size can grow over time, forming large inclusions. Our model does not account for cell size, and therefore no inferences can be made based off of syncytia size.

The other main limitation of our model is the lack of stochasticity. The use of an ODE assumes that the cells are continuous objects, but they are clearly discrete. Stochasticity can be introduced either through use of an ABM [58], which assumes that cells are discrete objects, or through the use of stochastic differential equations [59], which use a continuous time Markov chain assumption to change terms of the ODE to discrete events. The use of a continuous ODE model only allows for the model to represent the average dynamics of the entire assay, rather than being able to pick out individual interactions.

## 5. Conclusions

Despite these model limitations, our study has quantified the effect of AraC on syncytia formation. We agree with Lineberger that decreased expression of the envelope protein affects the extent of fusion, though our analysis indicates that changes in the density of the Env protein alter the kinetics of syncytia formation. We found that both syncytia formation and fusion duration decreased as AraC dosage increased. The more complex model introducing density dependence outperformed a basic model for syncytia formation in an AraC-treated cell–cell fusion assay. The two strains investigated here also showed significantly different density dependence, even at low dosages of AraC. This suggests that different strains of HIV have different responses to changes in surface density of glycoproteins, resulting in different syncytia formation dynamics.

## Figures and Tables

**Table 1:** Best-fit parameters for the basic model for HXB2.

Dosage	D(0)	γ (/min)	1/k (min)	SSR
10 M AraC	0.924	$4.16\times 10^{-3}$	1.02 $\times \;10^{-4}$	4.76 $\times \;10^{-4}$
95% CI	(0.889, 0.943)	(2.45, 7.30) $\times \;10^{-3}$	(0.00824, 16,300) $\times \;10^{-4}$	(3.67, 17.7) $\times \;10^{-4}$
320 nM AraC	0.871	$3.95\times 10^{-3}$	1.14 $\times \;10^{-4}$	2.59 $\times \;10^{-4}$
95% CI	(0.860, 0.880)	(3.49, 4.41) $\times \;10^{-3}$	(0.223, 3650) $\times \;10^{-4}$	(2.01, 2.61) $\times \;10^{-4}$
160 nM AraC	0.852	0.499	87.0	9.96 $\times \;10^{-4}$
95% CI	(0.847, 0.859)	(0.0583, 1.00)	(73.2, 93.7)	(7.07, 22.2) $\times \;10^{-4}$
80 nM AraC	0.817	0.0433	64.6	0.00205
95% CI	(0.813, 0.894)	(0.00847, 0.187)	(6.47 $\times \;10^{-5}$, 77.0)	(0.00139, 0.596)
40 nM AraC	0.807	0.0339	55.3	0.00397
95% CI	(0.799, 0.864)	(0.0168, 1.42)	(6.47 $\times \;10^{-5}$, 76.2)	(0.00287, 0.484)
20 nM AraC	0.804	0.0333	54.5	0.00417
95% CI	(0.796, 0.859)	(0.0158, 0.589)	(6.48 $\times \;10^{-5}$, 75.6)	(0.00286, 0.792)
10 nM AraC	0.804	0.0421	54.6	0.00416
95% CI	(0.799, 0.859)	(0.0103, 0.910)	(6.47 $\times \;10^{-5}$, 69.3)	(0.00291, 0.809)
5 nM AraC	0.799	0.0411	51.2	0.00484
95% CI	(0.793, 0.823)	(0.0182, 0.147)	(6.49 $\times \;10^{-5}$, 65.8)	(0.00335, 0.476)
2.5 nM AraC	0.796	0.0300	46.8	0.00508
95% CI	(0.790, 0.799)	(0.0254, 0.105)	(40.1, 67.6)	(0.00369, 0.00579)
0 nM AraC	0.791	0.0364	51.8	0.00552
95% CI	(0.784, 0.816)	(0.0186, 0.143)	(6.99, 53.7)	(0.00375, 0.502)

**Table 2:** Best-fit parameters for the basic model for Sf162.

Dosage	D(0)	γ (/min)	1/k (min)	SSR
10 μM AraC	0.833	$1.91\times 10^{-3}$	3.52 $\times \;10^{-6}$	0.00455
95% CI	(0.576, 0.840)	(0.610, 1.97) $\times \;10^{-3}$	(0.140, 21.3) $\times \;10^{-6}$	(0.00425, 0.00689)
2 μM AraC	0.831	$2.23\times 10^{-3}$	6.53 $\times \;10^{-6}$	0.00362
95% CI	(0.785, 0.834)	(1.59, 2.23) $\times \;10^{-3}$	(1.41, 25.3) $\times \;10^{-6}$	(0.00324, 0.00362)
750 nM AraC	0.859	$4.96\times 10^{-3}$	5.88 $\times \;10^{-6}$	0.0106
95% CI	(0.847, 0.887)	(4.33, 9.95) $\times \;10^{-3}$	(0.169, 176,000) $\times \;10^{-6}$	(0.00977, 0.0911)
500 nM AraC	0.857	$9.09\times 10^{-3}$	7.10 $\times \;10^{-6}$	0.03298
95% CI	(0.769, 0.864)	(3.32, 10.2) $\times \;10^{-3}$	(0.422, 152,000) $\times \;10^{-6}$	(0.0296, 0.190)
375 nM AraC	0.808	0.0147	8.79 $\times \;10^{-6}$	0.0925
95% CI	(0.804, 0.839)	(0.0131, 0.999)	(0.000544, 8.77) $\times \;10^{-6}$	(0.0847, 1.62)
250 nM AraC	0.788	0.0113	3.44	0.0826
95% CI	(0.781, 0.793)	(0.000983, 0.0131)	(0.133, 6.83)	(0.0746, 0.0826)
125 nM AraC	0.753	0.0184	2.06 $\times \;10^{-5}$	0.226
95% CI	(0.749, 0.759)	(0.0165, 0.0202)	(0.802, 46.1) $\times \;10^{-5}$	(0.207, 0.226)
62.5 nM AraC	0.734	0.0141	0.300	0.212
95% CI	(0.724, 0.735)	(0.0118, 0.0151)	(1.02 $\times \;10^{-5}$, 6.33)	(0.193, 0.232)
0 nM AraC	0.727	0.0144	2.99	0.219
95% CI	(0.720, 0.735)	(0.0126, 0.0178)	(0.00330, 6.40)	(0.198, 0.243)

**Table 3:** Density-dependent syncytia formation rate model best-fit parameters for HXB2.

Dosage	D(0)	γ (/min)	1/k (min)	α (/cell)	SSR
10 M AraC	0.165	$5.92\times 10^{-4}$	2.73	176	2.94 $\times \;10^{-4}$
95% CI	(0.162, 0.169)	(5.75, 60.8) $\times \;10^{-4}$	(6.08 $\times \;10^{-4}$, 6.29)	(167, 185)	(2.27, 2.99) $\times \;10^{-4}$
320 nM AraC	0.119	$7.84\times 10^{-4}$	3.43	104	1.97 $\times \;10^{-4}$
95% CI	(0.118, 0.121)	(7.12, 8.21) $\times \;10^{-4}$	(0.880, 5.29)	(83.8, 115)	(1.52, 2.32) $\times \;10^{-4}$
160 nM AraC	0.779	$5.69\times 10^{-3}$	19.9	4.08	9.35 $\times \;10^{-4}$
95% CI	(0.0562, 0.783)	(1.17, 8.80) $\times \;10^{-3}$	(2.69 $\times \;10^{-7}$, 21.2)	(1.78, 417)	(6.57, 36.9) $\times \;10^{-4}$
80 nM AraC	0.795	$1.68\times 10^{-2}$	42.1	5.57	1.92 $\times \;10^{-3}$
95% CI	(2.70 $\times \;10^{-4}$, 0.815)	(0.0959, 36.5) $\times \;10^{-2}$	(6.29 $\times \;10^{-8}$, 48.0)	(1.84 $\times \;10^{-4}$, 16,300)	(1.47, 12.4) $\times \;10^{-3}$
40 nM AraC	0.790	$2.04\times 10^{-2}$	43.0	4.89	3.85 $\times \;10^{-3}$
95% CI	(8.89 $\times \;10^{-4}$, 0.808)	(0.115, 62.3) $\times \;10^{-2}$	(8.86 $\times \;10^{-8}$, 49.9)	(7.72 $\times \;10^{-10}$, 13,800)	(2.85, 184) $\times \;10^{-3}$
20 nM AraC	0.791	$2.15\times 10^{-2}$	43.7	3.93	4.09 $\times \;10^{-3}$
95% CI	(2.44 $\times \;10^{-4}$, 0.804)	(0.121, 57.2) $\times \;10^{-2}$	(7.46 $\times \;10^{-7}$, 69.4)	(1.28 $\times \;10^{9}$, 8530)	(2.92, 333) $\times \;10^{-3}$
10 nM AraC	0.785	$2.17\times 10^{-2}$	39.7	5.55	3.91 $\times \;10^{-3}$
95% CI	(5.43 $\times \;10^{-4}$, 0.802)	(0.137, 74.7) $\times \;10^{-2}$	(7.21 $\times \;10^{-8}$, 46.9)	(0.608, 15,200)	(2.82, 147) $\times \;10^{-3}$
5 nM AraC	0.783	$2.33\times 10^{-2}$	38.5	4.69	4.59 $\times \;10^{-3}$
95% CI	(6.50 $\times \;10^{-4}$, 0.798)	(0.153, 99.9) $\times \;10^{-2}$	(6.11 $\times \;10^{-8}$, 48.3)	(2.76 $\times \;10^{-5}$, 13,600)	(3.31, 181) $\times \;10^{-3}$
2.5 nM AraC	0.755	$1.94\times 10^{-2}$	38.4	9.51	4.08 $\times \;10^{-3}$
95% CI	(4.02 $\times \;10^{-4}$, 0.784)	(0.138, 55.5) $\times \;10^{-2}$	(7.10 $\times \;10^{-8}$, 41.2)	(2.54, 11,300)	(2.98, 22.7) $\times \;10^{-3}$
0 nM AraC	0.778	$2.35\times 10^{-2}$	41.2	3.42	5.41 $\times \;10^{-3}$
95% CI	(0.00154, 0.789)	(0.152, 77.3) $\times \;10^{-2}$	(1.12 $\times \;10^{-7}$, 46.8)	(2.78 $\times \;10^{-9}$, 13,700)	(3.79, 204) $\times \;10^{-3}$

**Table 4:** Density-dependent syncytia formation rate model best-fit parameters for Sf162.

Dosage	D(0)	γ (/min)	1/k (min)	α (/cell)	SSR
10 μM AraC	3.51 $\times \;10^{-4}$	$3.67\times 10^{-4}$	0.340	17,700	3.94 $\times \;10^{-3}$
95% CI	(3.33, 4.01) $\times \;10^{-4}$	(3.57, 4.00) $\times \;10^{-4}$	(0.0857, 7.54)	(16,200, 21,500)	(3.69, 4.93) $\times \;10^{-3}$
2 μM AraC	1.87 $\times \;10^{-4}$	$4.30\times 10^{-4}$	0.0368	31,700	2.97 $\times \;10^{-3}$
95% CI	(1.73, 2.06) $\times \;10^{-4}$	(4.25, 4.66) $\times \;10^{-4}$	(0.0251, 0.0537)	(28,500, 37,900)	(2.77, 3.17) $\times \;10^{-3}$
750 nM AraC	7.41 $\times \;10^{-3}$	$1.49\times 10^{-3}$	9.16	3650	4.15 $\times \;10^{-3}$
95% CI	(7.34, 7.59) $\times \;10^{-3}$	(1.43, 1.58) $\times \;10^{-3}$	(6.69, 11.5)	(3500, 4360)	(3.87, 4.18) $\times \;10^{-3}$
500 nM AraC	0.0415	$7.09\times 10^{-3}$	5.92	2380	5.23 $\times \;10^{-3}$
95% CI	(0.0369, 0.0455)	(6.16, 8.14) $\times \;10^{-3}$	(4.95, 7.03)	(1990, 2820)	(4.66, 5.22) $\times \;10^{-3}$
375 nM AraC	0.0497	$2.46\times 10^{-2}$	9.15	3870	0.0621
95% CI	(0.0480, 0.080)	(2.28, 26.5) $\times \;10^{-2}$	(0.945, 9.72)	(3310, 12,700)	(5.56, 92.5) $\times \;10^{-3}$
250 nM AraC	0.0320	$9.78\times 10^{-3}$	18.8	2290	0.0184
95% CI	(0.0298, 0.0354)	(9.24, 14.4) $\times \;10^{-3}$	(16.5, 20.6)	(2010, 3250)	(0.0165, 0.0381)
125 nM AraC	0.0653	$5.56\times 10^{-2}$	9.49	4020	6.15 $\times \;10^{-3}$
95% CI	(0.0643, 0.0685)	(5.25, 5.84) $\times \;10^{-2}$	(9.11, 9.86)	(3610, 4300)	(5.49, 6.15) $\times \;10^{-3}$
62.5 nM AraC	0.0451	$2.05\times 10^{-2}$	19.8	2210	0.0518
95% CI	(0.0410, 0.0510)	(1.87, 2.37) $\times \;10^{-2}$	(17.6, 21.4)	(1860, 2960)	(0.0464, 0.0526)
0 nM AraC	0.0682	$2.27\times 10^{-2}$	19.3	1470	0.0493
95% CI	(0.0417, 0.0789)	(2.13, 31.8) $\times \;10^{-2}$	(1.43, 20.7)	(1250, 15,900)	(0.0443, 0.368)

**Table 5:** AIC values for both model fits to the experimental data.

HXB2			Sf162		
**Dosage**	**Basic AIC**	**DD AIC**	**Dosage**	**Basic AIC**	**D.D AIC**
10 M	−219	**−227**	10 μM	−701	**−709**
320 nM	−231	**−235**	2 μM	−641	**−653**
160 μM	**−203**	−202	750 nM	−639	**−706**
80 nM	**−188**	−187	500 nM	−513	**−636**
40 nM	**−175**	−173	375 nM	−423	**−625**
20 nM	**−173**	−171	250 nM	−489	**−597**
10 nM	**−173**	−172	125 nM	−382	**−626**
5 nM	**−170**	−169	62.5 nM	−420	**−521**
2.5 nM	−169	**−171**	0 nM	−411	**−517**
0 nM	**−167**	−166			

## Data Availability

All data are included within the manuscript.

## Supplementary Materials

The following supporting information can be downloaded at: https://www.mdpi.com/article/10.3390/v17040583/s1. The supplementary file contains corner plots for all model fits.
