# Supplementary material for: Quantifying Impact of HIV Receptor Surface Density Reveals Differences in Fusion Dynamics of HIV Strains
Source: Viruses. 2025 Apr 18;17(4):583. doi: 10.3390/v17040583 (PMC12031222; doi:10.3390/v17040583)
Supplement: Supplementary file 1 [file viruses-17-00583-s001.zip › viruses-3439885-supplementary.pdf]

Supplement for "Quantifying the effect of HIV receptor surface density reveals differences in fusion dynamics of HIV strains"

Anthony Gerg and Hana M. Dobrovolny

1 Parameter histograms

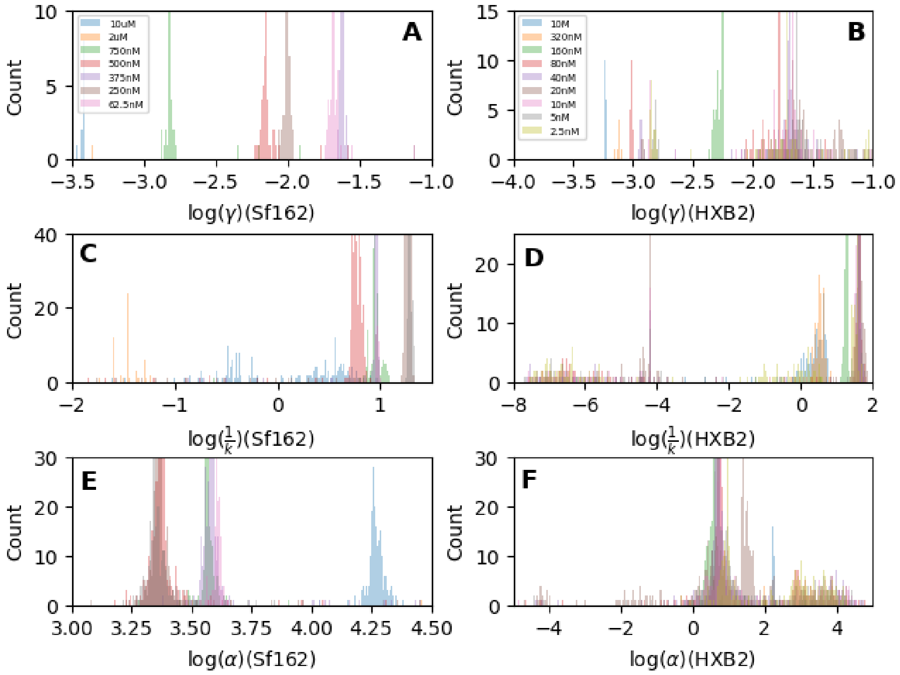

Figure S1: Histograms of bootstrap parameters for the density dependent model. Sf162 (left column) and HXB2 (right column). Figures show parameter distributions for (A,B) syncytia formation rate, γ, (C,D) fusion duration, $1/k$, and (E,F) density dependence, α.

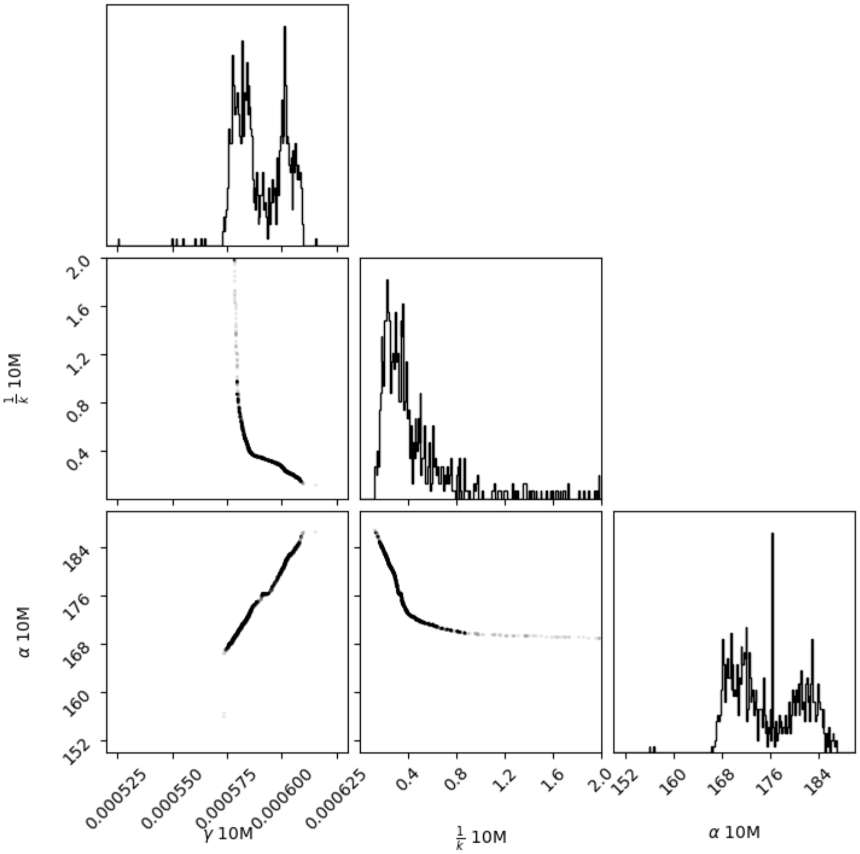

Figure S2: Corner plot for 10 M bootstrap data

2 Corner Plots

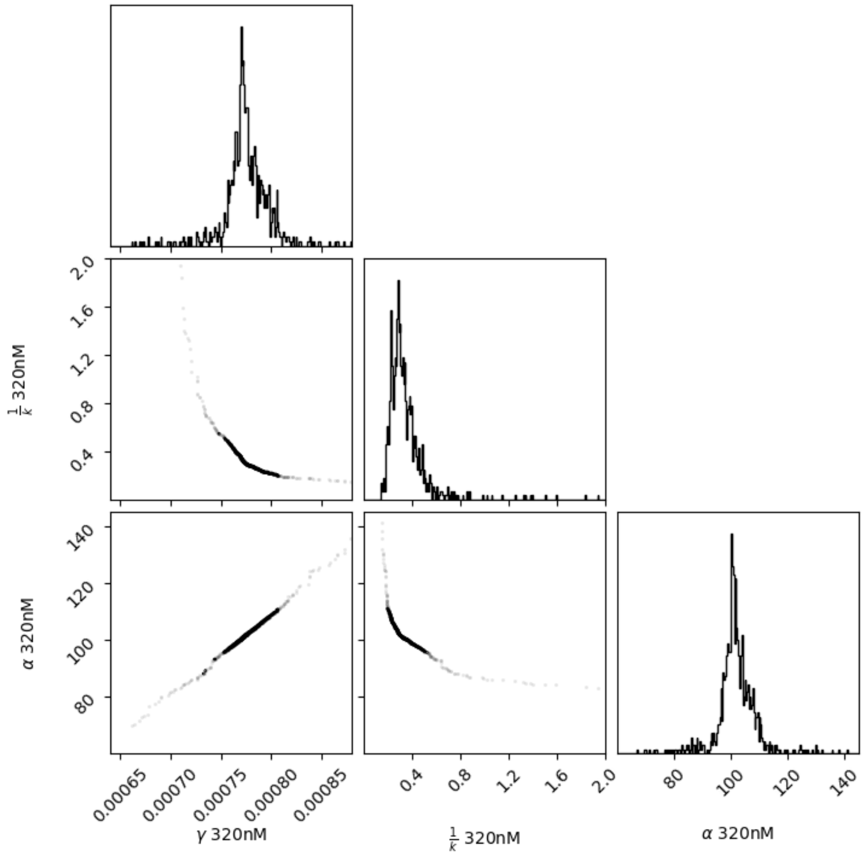

Figure S3: Corner plot for 320 nM bootstrap data

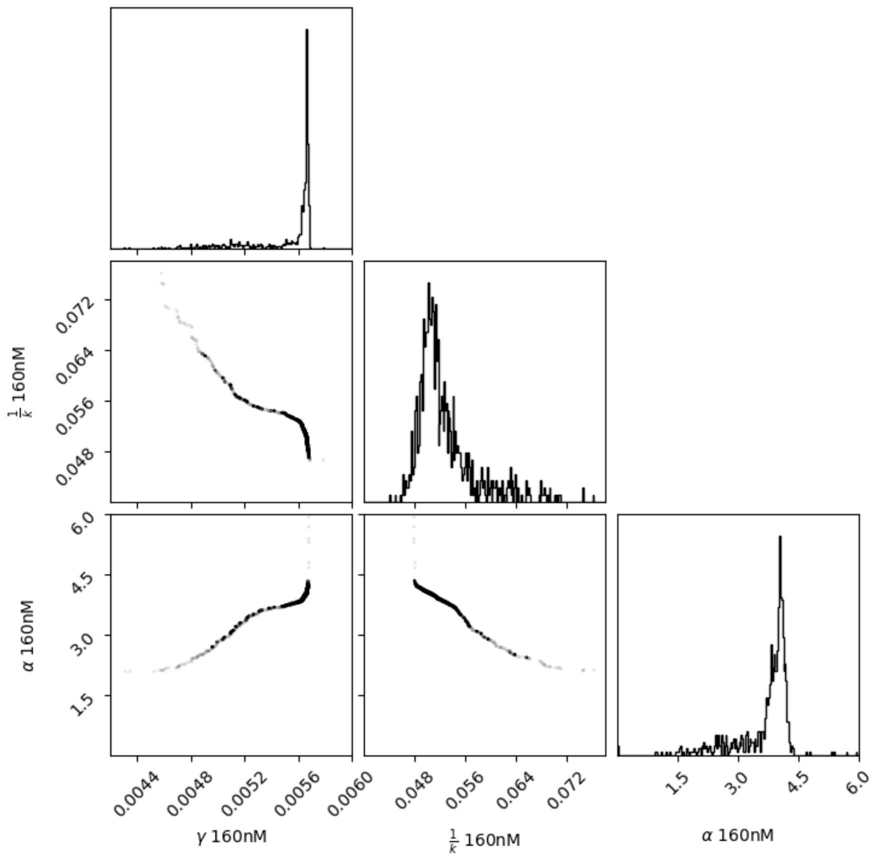

Figure S4: Corner plot for 160 nM bootstrap data

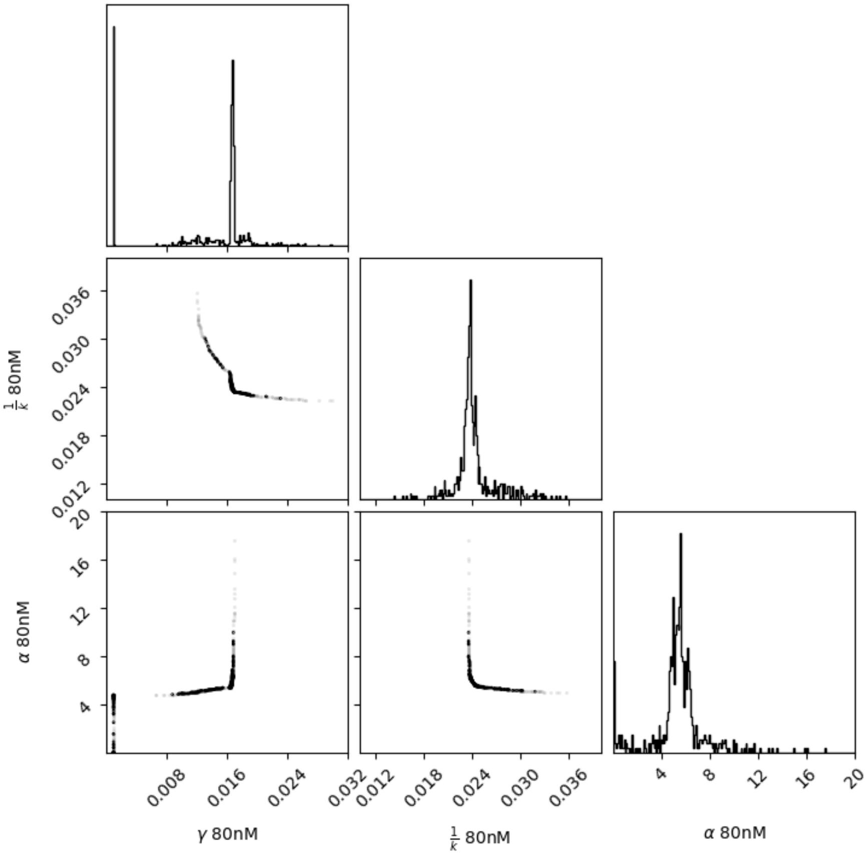

Figure S5: Corner plot for 80 nM bootstrap data

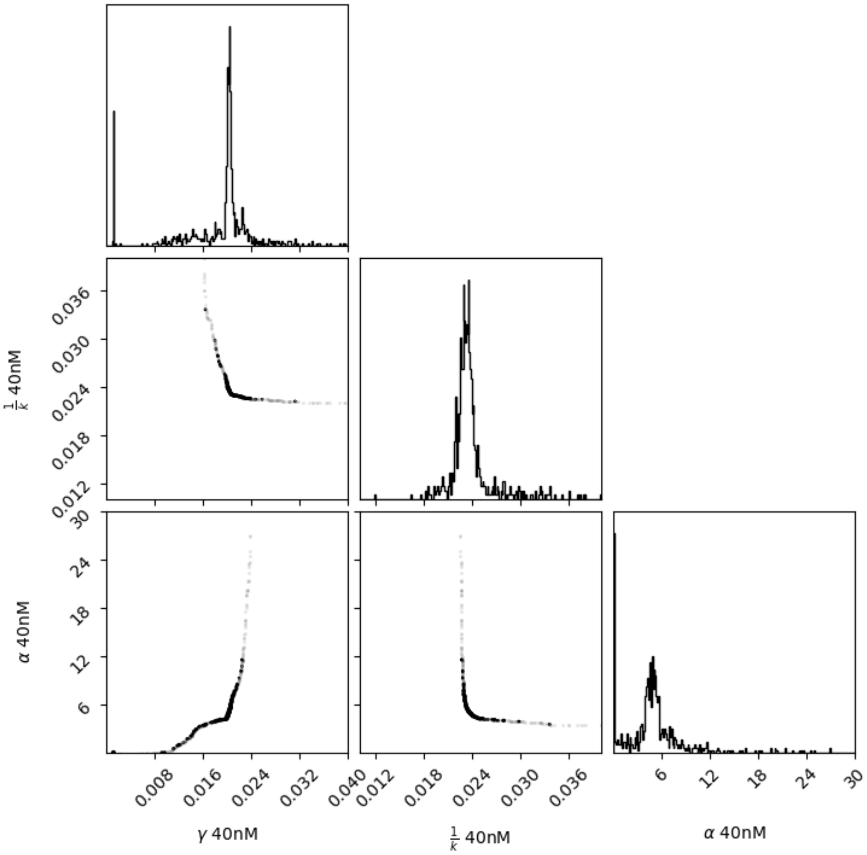

Figure S6: Corner plot for 40 nM bootstrap data

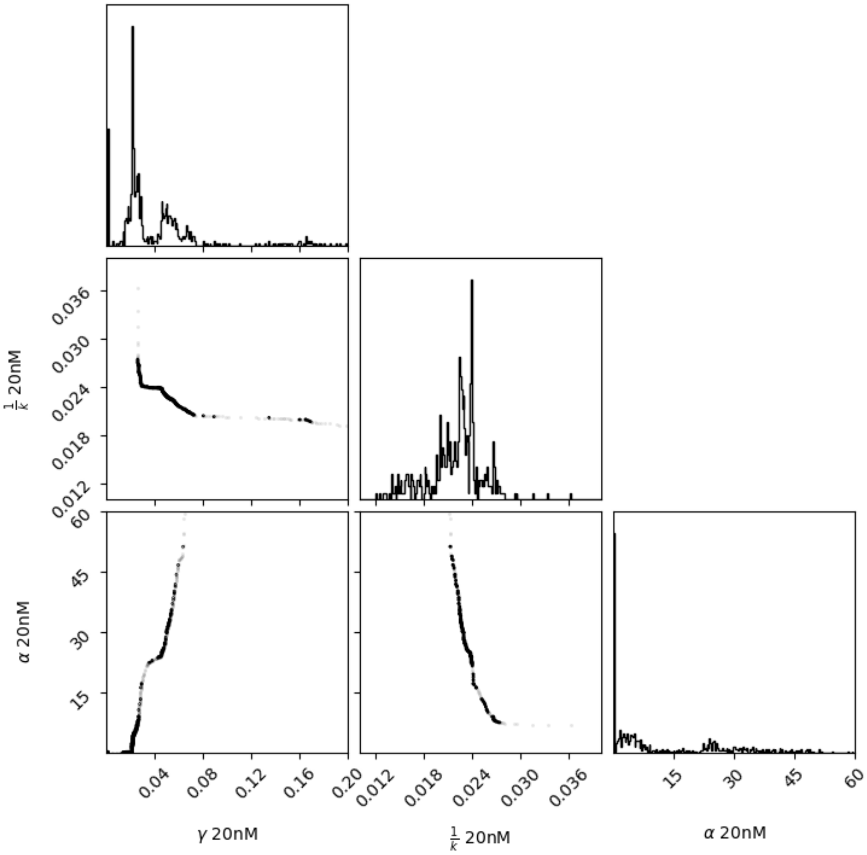

Figure S7: Corner plot for 20 nM bootstrap data

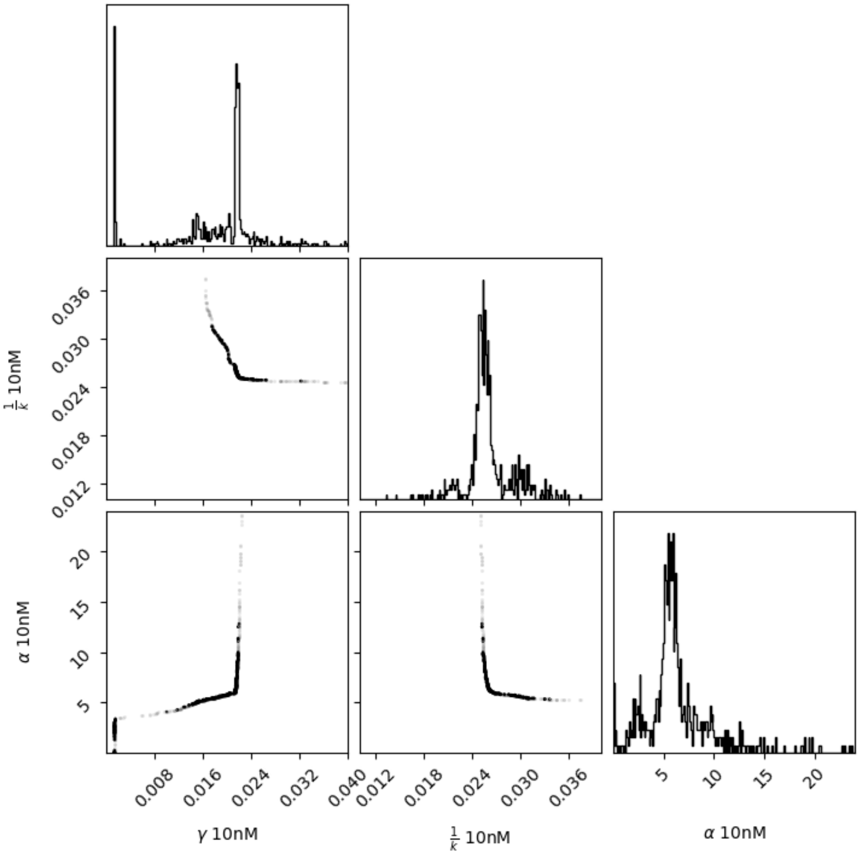

Figure S8: Corner plot for 10 nM bootstrap data

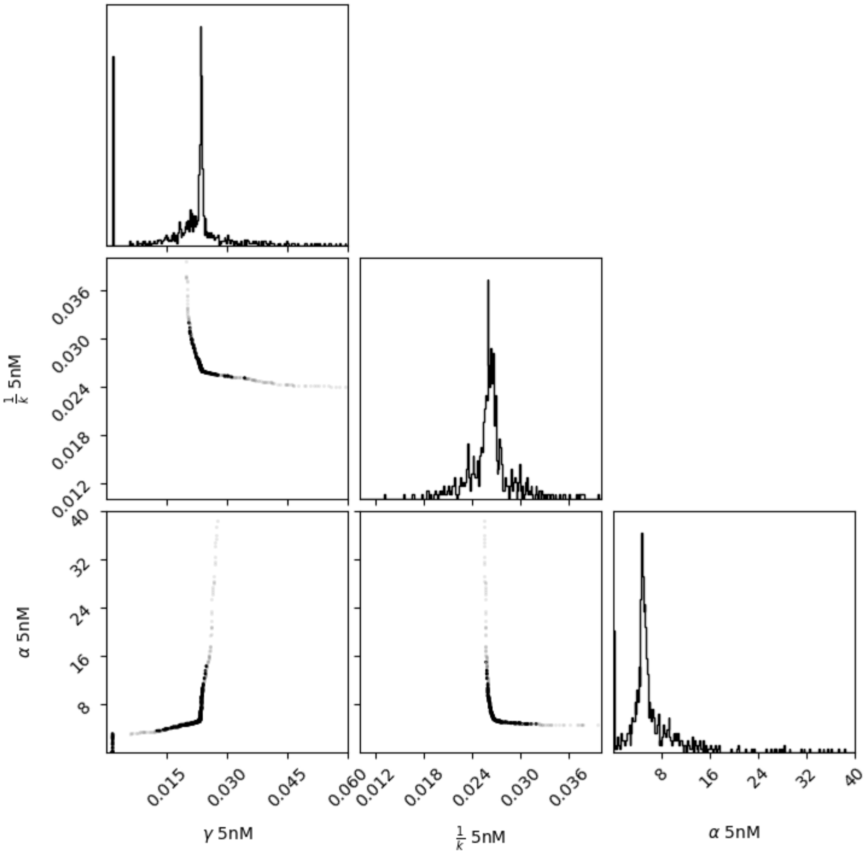

Figure S9: Corner plot for 5 nM bootstrap data

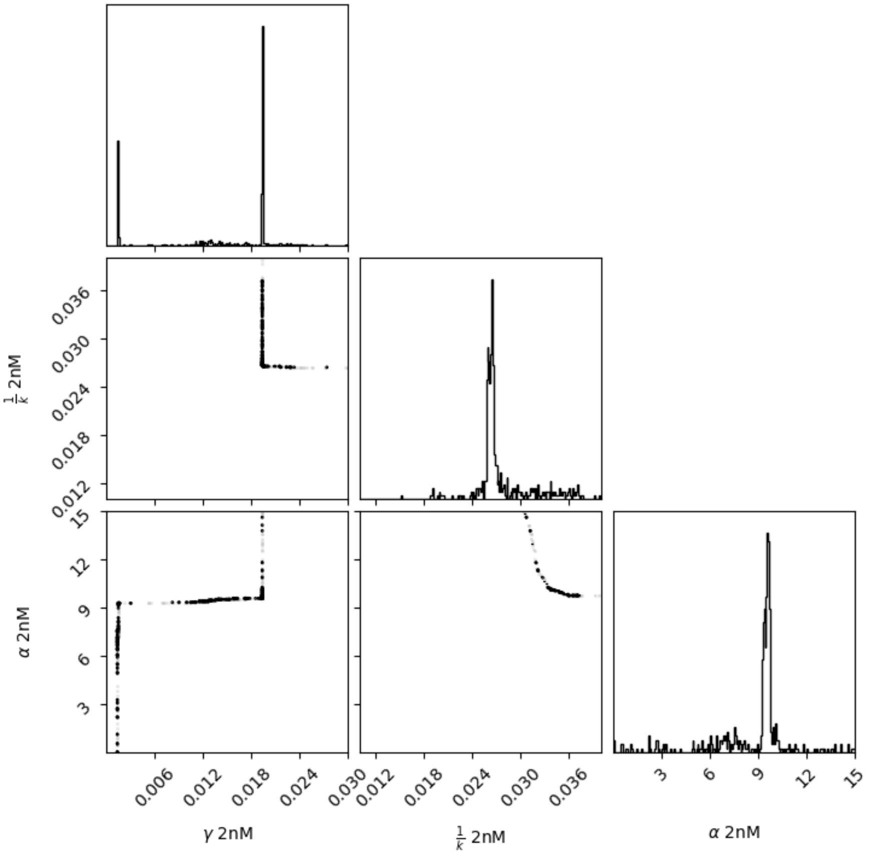

Figure S10: Corner plot for 2.5 nM bootstrap data

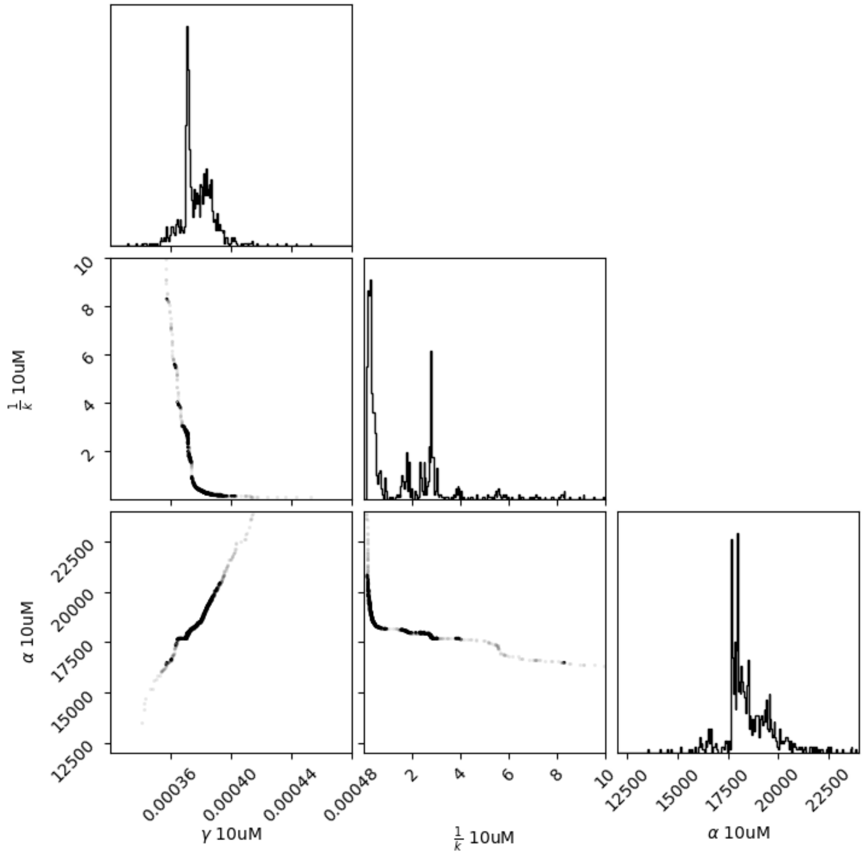

Figure S11: Corner plot for 10 μM bootstrap data

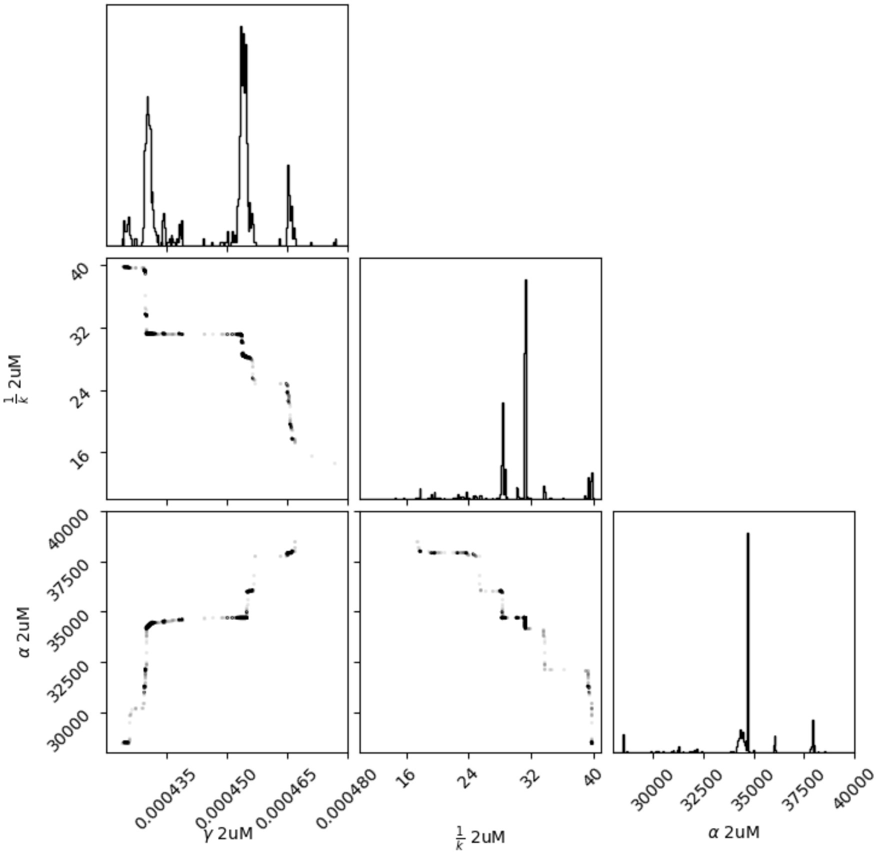

Figure S12: Corner plot for 2 μM bootstrap data

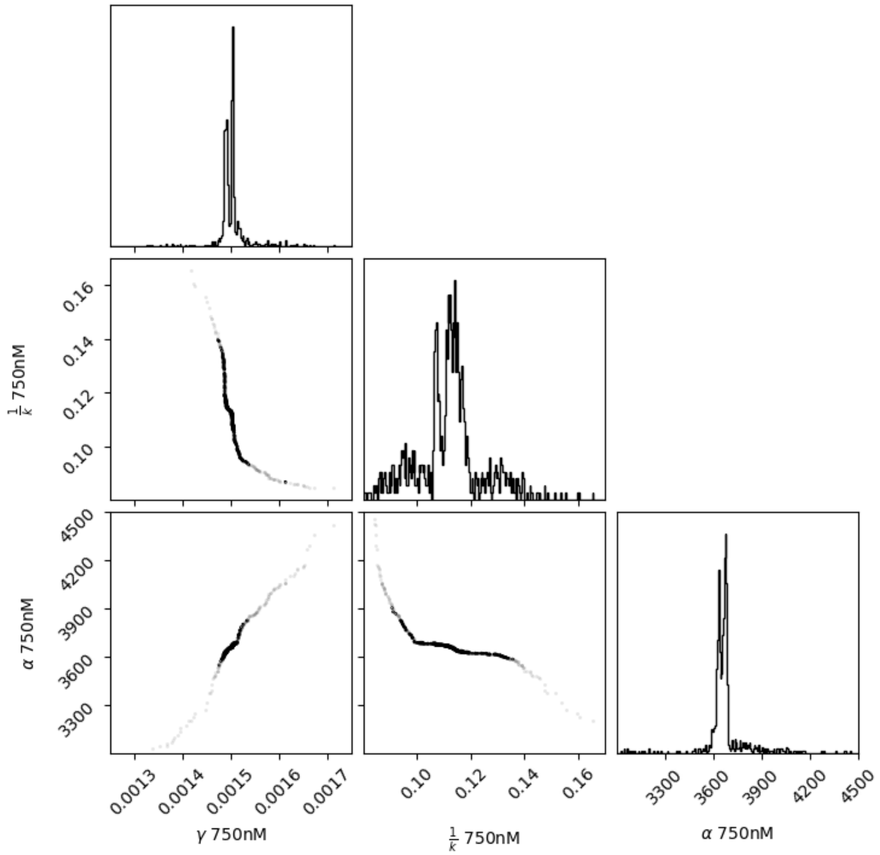

Figure S13: Corner plot for 750 nM bootstrap data

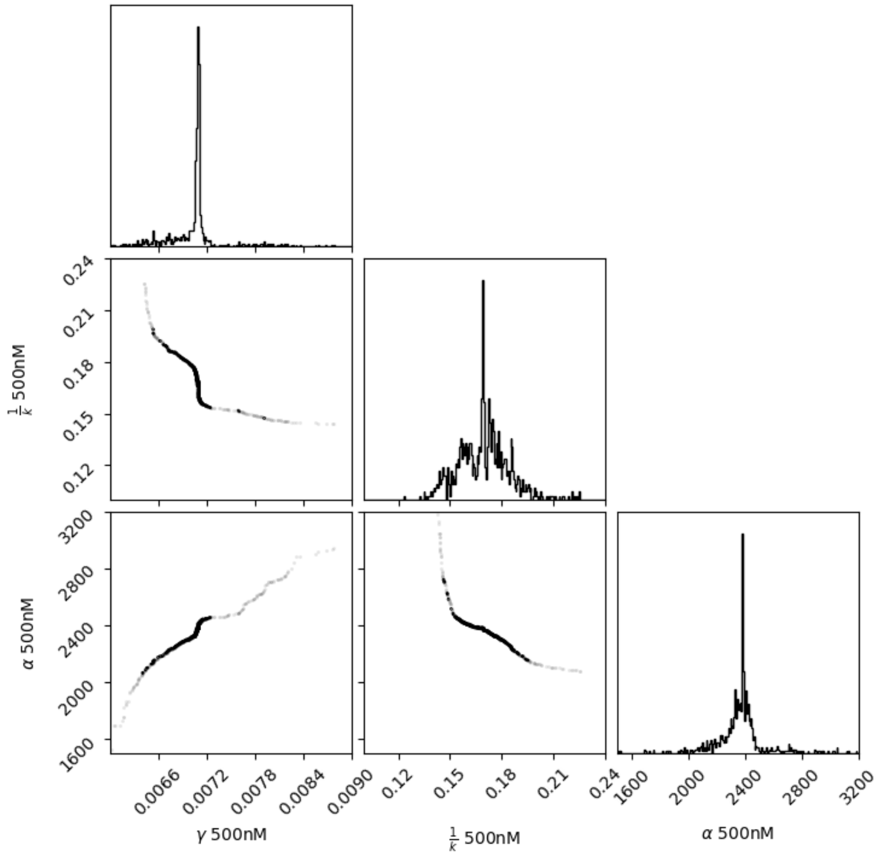

Figure S14: Corner plot for 500 nM bootstrap data

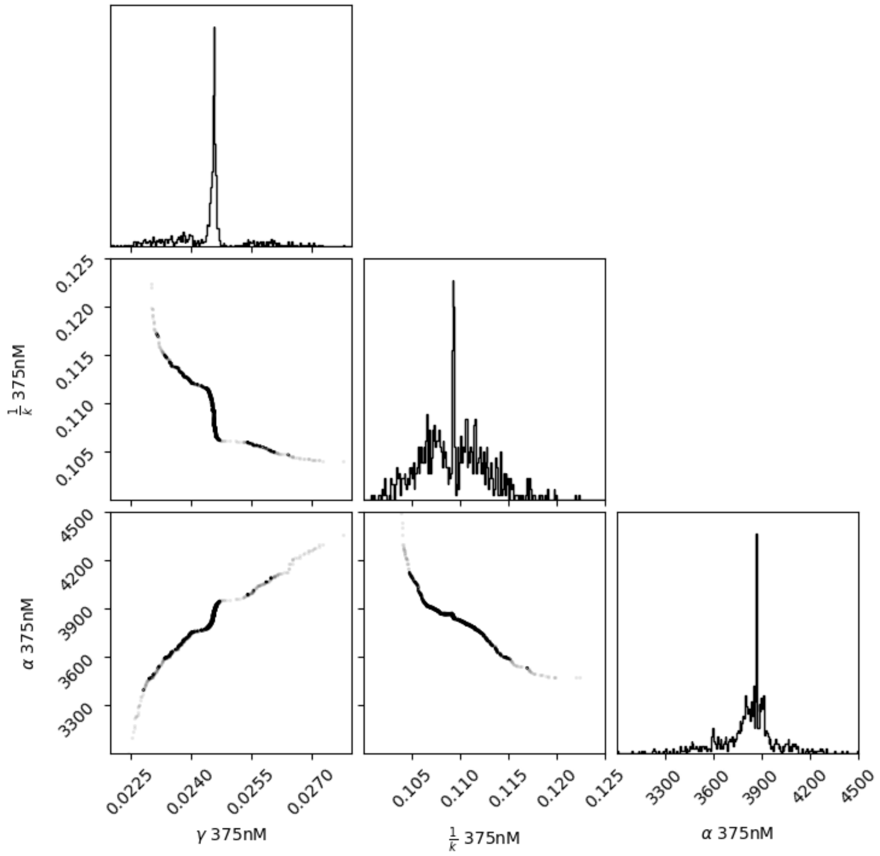

Figure S15: Corner plot for 375 nM bootstrap data

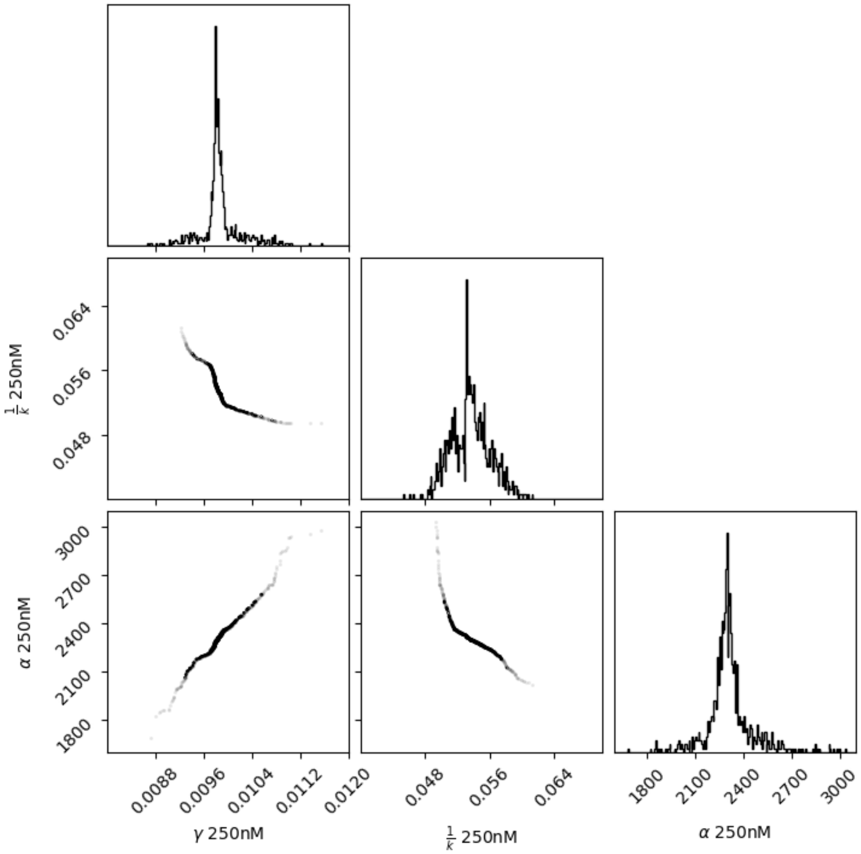

Figure S16: Corner plot for 250 nM bootstrap data

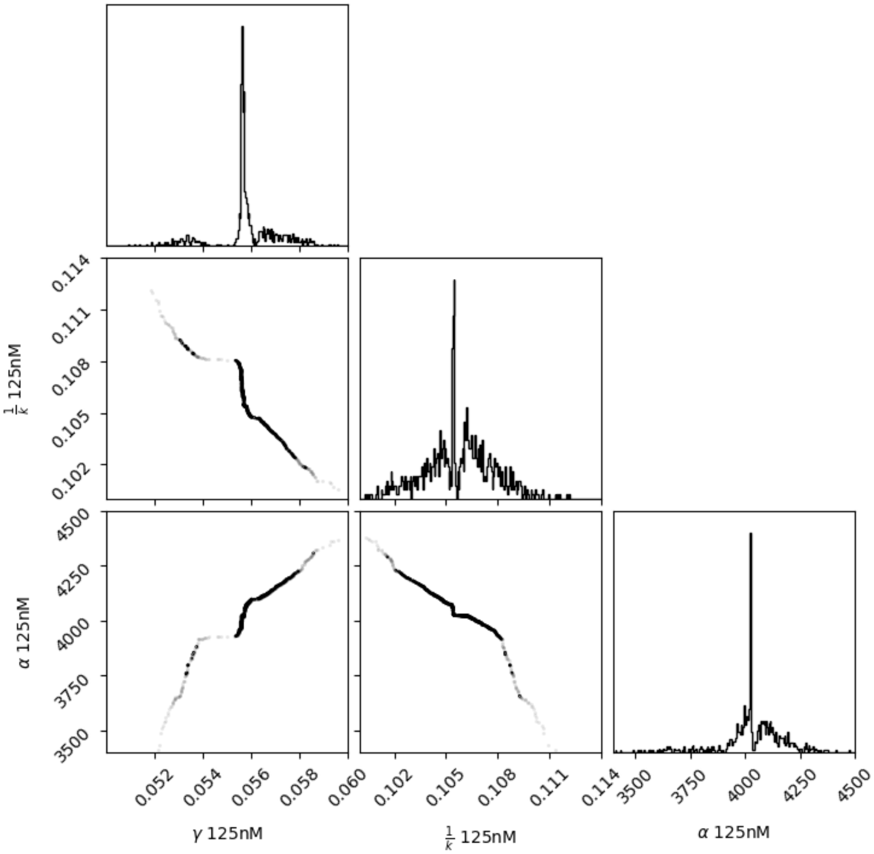

Figure S17: Corner plot for 125 nM bootstrap data

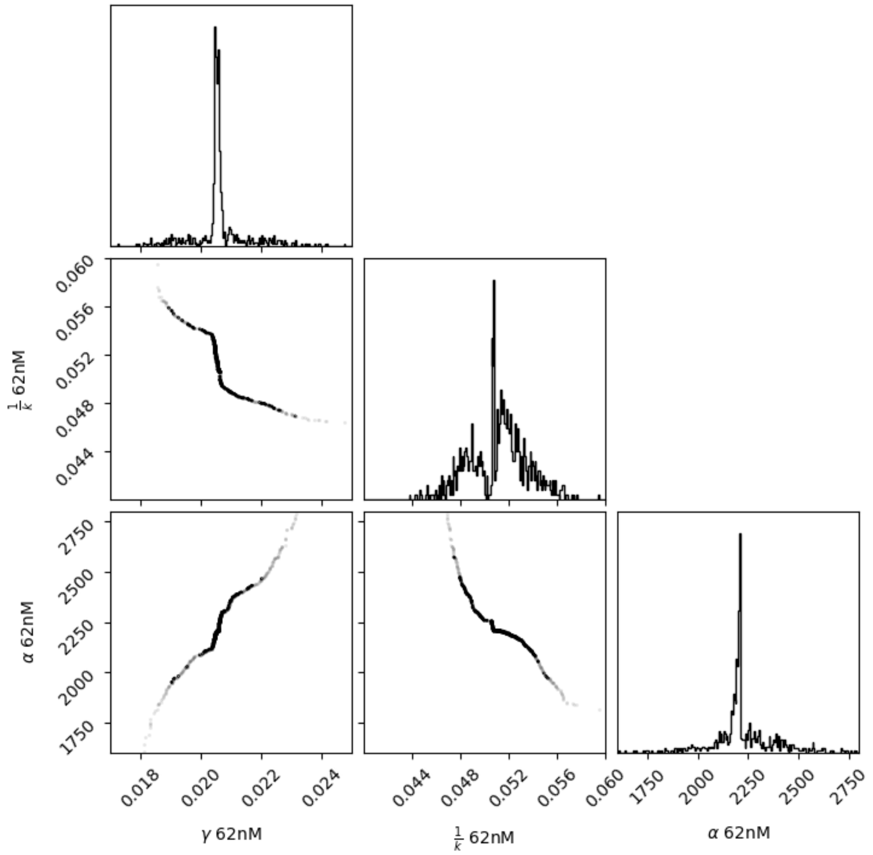

Figure S18: Corner plot for 62.5 nM bootstrap data
